# Phytoecdysteroids Do Not Have Anabolic Effects in Skeletal Muscle in Sedentary Aging Mice

**DOI:** 10.3390/ijerph18020370

**Published:** 2021-01-06

**Authors:** Marcus M. Lawrence, Kevin A. Zwetsloot, Susan T. Arthur, Chase A. Sherman, Joshua R. Huot, Vladimir Badmaev, Mary Grace, Mary Ann Lila, David C. Nieman, R. Andrew Shanely

**Affiliations:** 1 Department of Health and Exercise Science, Appalachian State University, Boone, NC 28608, USA; marcuslawrence@suu.edu (M.M.L.); zwetslootka@appstate.edu (K.A.Z.); casherman0412@email.campbell.edu (C.A.S.); 2 Human Performance Laboratory, North Carolina Research Campus, Kannapolis, NC 28081, USA; niemandc@appstate.edu; 3 Integrated Muscle Physiology Laboratory, Boone, NC 28607, USA; 4 Department of Kinesiology and Outdoor Recreation, Southern Utah University, Cedar City, UT 84720, USA; 5 Laboratory of Systems Physiology, Department of Kinesiology, University of North Carolina Charlotte, Charlotte, NC 28223, USA; sarthur8@uncc.edu (S.T.A.); jrhuot@iu.edu (J.R.H.); 6 Department of Biology, Appalachian State University, Boone, NC 20608, USA; 7 American Medical Holdings Inc., Staten Island, NY 10314, USA; vebadmaev@attglobal.net; 8 Plants for Human Health Institute, North Carolina Research Campus, North Carolina State University, Kannapolis, NC 28081, USA; mhgrace@ncsu.edu (M.G.); maryann_lila@ncsu.edu (M.A.L.)

**Keywords:** skeletal muscle, hypertrophy, Akt, p70S6K, atrogenes, 20-hydroxyecdysone

## Abstract

Skeletal muscle mass and strength are lost with aging. Phytoecdysteroids, in particular 20-hydroxyecdysone (20E), increase protein synthesis in C2C12 skeletal muscle cells and muscle strength in young rats. The objective of this study was to determine whether an extract from *Ajuga turkestanica* (ATE), enriched in phytoecdysteroids, and 20E affect skeletal muscle mass and fiber size, fiber type, activation of the PI3K–Akt signaling pathway, and the mRNA levels of *MAFbx*, *MuRF-1*, and *myostatin* in sedentary aging mice. Aging male C57BL/6 mice (20 months old) received ATE, 20E, or vehicle (CT) once per day for 28 days or a single acute dose. Treatment did not alter body, muscle, or organ mass; fiber cross-sectional area; or fiber type in the triceps brachii or plantaris muscles. Likewise, protein synthesis signaling markers (i.e., phosphorylation of Akt^Ser473^ and p70S6k^Thr389^) measured after either 28 days or acutely were unchanged. Neither ATE nor 20E treatment for 28 days affected the mRNA levels of *MAFbx*, *MuRF-1*, and *myostatin*. In conclusion, these data indicate that phytoecdysteroid treatment does not alter muscle mass or fiber type, nor does it activate protein synthesis signaling in the skeletal muscle of sedentary aging mice.

## 1. Introduction

The loss of muscle mass and strength with advanced age (sarcopenia) is associated with decreased physical function, including loss of independence and eventually premature death [1]. Muscle mass is controlled by the intricate balance of muscle protein synthesis and muscle protein degradation. Aged skeletal muscle has a blunted ability to stimulate muscle protein synthesis in response to anabolic stimuli, termed anabolic resistance [2]. The blunted muscle protein synthesis response observed in aged muscle is largely due to impaired activation of the PI3K/Akt/mTOR/p70S6K signaling axis or PI3K–Akt pathway [3,4]. However, current interventions to mitigate sarcopenia offer limited success.

Sarcopenia is also related to changes in the levels of negative growth regulators, e.g., myostatin, and muscle protein degradation enzymes. Myostatin limits skeletal muscle growth, by attenuating the PI3K–Akt pathway, thereby downregulating muscle protein synthesis [5,6]. Compared to young adult muscle, myostatin expression is elevated in aged humans, potentially contributing to the progression of sarcopenia [7]. Myofibrillar proteins, including actin and myosin, and many other structural proteins, are substrates for the ubiquitin–proteasome pathway, a critical muscle protein degradation pathway [8]. Two key enzymes involved in tagging proteins for degradation via the ubiquitin–proteasome pathway are the E3 ligases: muscle atrophy F-box (MAFbx)/atrogin-1 and muscle RING finger protein 1 (MuRF-1) [9]. Both enzymes have been reported to be increased in aged muscle [10] and are robust markers of muscle atrophy [9]. Additionally, MAFbx and MuRF-1 are negatively regulated by Akt in a forkhead box (FOXO)-dependent fashion [8]. Thus, Akt not only regulates protein synthesis via phosphorylation of mTOR and its downstream targets, e.g., p-70S6K1, but it also phosphorylates the FOXO transcription factors on conserved residues, Thr^24/32^, Ser^256/235^, and Ser^319/315^ [11,12]. FOXO inactivation prevents its nuclear translocation and activation of genes associated with protein degradation, e.g., MAFbx and MuRF-1 [8,13,14]. Inhibiting myostatin expression in aged mice is reported to decrease *MAFbx* mRNA levels, but not *MuRF-1*, and attenuate the loss of muscle mass [15].

*Ajuga turkestanica* (Regel) Briq. (Labiatae, Mint) is a wild plant native to Uzbekistan and other regions in Central Asia and contains an array of bioactive phytochemicals, in particular phytoecdysteroids [16]. Phytoecdysteroids are plant-produced analogues of ecdysteroids, insect molting hormones that control cell proliferation, growth, and development [17]. The phytoecdysteroids from *A. turkestanica* are reported to have anabolic, analgesic, anti-inflammatory, antihypertensive, antioxidant, antibacterial, and hepatoprotective properties [18,19]. Work from Gorelick-Feldman et al. has demonstrated that an extract from *A. turkestanica*, enriched in phytoecdysteroids, stimulates protein synthesis via the PI3K–Akt pathway in C2C12 skeletal muscle myotubes in vitro, and chronic 20-hydroxyecdysone (20E) treatment increases grip strength in young adult rats [20]. Zubeldia et al. reported that an extract from *A. turkestanica* decreased myostatin mRNA expression fourfold after treatment in myotubes in vitro [21]. These data suggest that *A. turkestanica* may increase PI3K–Akt signaling and may inhibit the expression of myostatin, MAFbx, and MuRF-1 in vivo. In other work, continuous administration of purified 20E to young adult male C57BL/6J mice for 5 days resulted in significant increases in the mass of the triceps brachii, although longer administration (15 days) showed no effect [22].

The efficacy of phytoecdysteriods to combat sarcopenia in aging skeletal muscle is currently unknown. Therefore, the purpose of this study was to determine the extent to which feeding an extract from *A. turkestanica* or a single phytoecdysteroid (20E) to sedentary aging mice would activate the key control point of protein synthesis, the PI3K–Akt pathway, and attenuate the mRNA levels of the negative growth regulators, MAFbx, MuRF-1, and myostatin.

## 2. Materials and Methods

### 2.1. Plant Materials and Crude Extraction

Whole *A. turkestanica* plant material was harvested in Las Palmas, Spain (PoliNat; Las Palmas, Spain). The plant material was air dried and milled to 1–2 mm particle size. A crude extraction, consisting of a 30:70 water–ethanol solvent solution at 60 °C to extract phytoecdysteroids and sterilize the plant material, was performed by P.L. Thomas Inc. (Morristown, NJ, USA); P.L. Thomas Inc. kindly donated this initial extract. HPLC analysis of the sterilized and extracted *A. turkestanica* plant material, normalized to a 20E standard, revealed a 6% total phytoecdysteroid content in the initial crude extract. The initial extract was air dried and sieved through a 60 mm mesh screen and stored at −20 °C until further extraction and analysis. The extract enriched in phytoecdysteroids from the initial *A. turkestanica* extract (ATE) was prepared and quantified using established methods [16]. (see online Supplementary Materials).

### 2.2. Animals

#### 2.2.1. Chronic Treatment

Aging male C57BL/6 mice, 20-months-old (equivalent to ~60 human years [23]), were obtained from the National Institutes on Aging (NIA) Aged Rodent Colony. Based on the outcome measure of changes in grip strength in a previous study [20] a minimal total sample size of 33, 11 per group, was determined by an a priori power analysis (G*Power, Dusseldorf, Germany) for F-tests of one-way ANOVA (f = 0.589; 1-β = 0.80, alpha = 0.05). To account for potential loss due to aging, a total of 36 mice were used in the study. Mice were randomly assigned to one of three treatment groups: vehicle (CT; *n* = 12), ATE (50 mg/kg/day; *n* = 12), or 20E (50 mg/kg/day; *n* = 12). The dose and treatment period of 28 days is based on the previous study by Gorelick-Feldman et al. [20]. ATE and 20E (E6425-HE; Bosche Scientific, New Brunswick, NJ, USA) were suspended in a vehicle (100% non-denatured ethanol; EtOH; Control, CT. At the end of each light cycle (at 08:00 hours), body mass was measured. The previous day’s body mass was used to calculate the absolute amount of ATE, 20E, or vehicle needed to achieve the 50 mg/kg daily dose for each animal. The ATE, 20E, or vehicle (EtOH) was aliquoted onto one 190 mg rodent enrichment treat (Fruit Crunchies; BioServ, Frenchtown, NJ, USA) the day before feeding it to the animal in order to allow the EtOH to evaporate. Mice were placed individually in an empty cage without bedding and fed one treated Fruit Crunchie per day for 28 days. Complete consumption of the dosed Fruit Crunchie was visually confirmed for each mouse. Twenty-four hours after the final treatment, mice were euthanized while under inhaled isoflurane. Skeletal muscles and organs (including: heart, liver, spleen, kidneys, and testes) were quickly dissected and wet weight mass was recorded. The plantaris, triceps brachii, and gastrocnemius were selected for study as they are composed of predominantly type II muscle fibers and type II muscle fibers, as opposed to type I fibers, are preferentially prone to sarcopenia in both humans [24,25] and rodents [26,27]. Skeletal muscles and organs were immediately snap frozen in liquid N_2_ or liquid N_2_-cooled isopentane for histology (plantaris and triceps brachii) and stored at −80 °C in a freezer until subsequent analysis.

#### 2.2.2. Acute Treatment 

Aging male C57BL/6J mice (NIA Aged Rodent Colony), *n* = 20, 20-month-old, were randomly assigned to one of three treatment groups and given a single oral dose (via gavage) of either ATE (*n* = 7; 50 mg/kg) or 20E (*n* = 7; 50 mg/kg) or vehicle (corn oil; CT; *n* = 6). Mice were euthanized under inhaled isoflurane 90 min after acute treatment and skeletal muscles were immediately harvested, snap frozen in liquid nitrogen, and stored at −80 °C until further analysis.

Mice were housed within the David H. Murdock Research Institute (DHMRI) Center for Laboratory Animal Sciences at the North Carolina Research Campus, Kannapolis, NC. The room was maintained at 21 °C, 50% humidity, with a 12:12 h reverse light cycle. Food and water were provided ad libitum. All experiments were approved by the North Carolina Research Campus Institutional Animal Care and Use Committee and the experiments were conducted within the guidelines set forth by the American Physiological Society.

### 2.3. Myosin Heavy Chain Fiber Typing Immunofluorescence Analysis

Plantaris and triceps brachii muscles were mounted on cork in a tragacanth gum/optimal cutting temperature (OCT; Thermo Fisher Scientific Inc., Waltham, MA, USA) mixture and frozen in liquid N_2_-cooled isopentane for histological analyses. Muscles were then cut into 10 μm cross-sections using a cryoSTAT (Model Microm HM 505; Thermo Fisher Scientific Inc.). Myosin heavy chain (MHC) fiber type immunofluorescent detection was performed using published methods [28], with modifications. Briefly, muscles were blocked and incubated overnight with a primary antibody cocktail containing MHCIIA (Developmental Studies Hybridoma Bank: DHSB #SC-71), or MHCIIB (DSHB #BF-F3), or Laminin (Millipore #05-206; Bradford, MA, USA). Type I MHC was not assessed as the plantaris does not express type I fibers and type I expression is less than 3% in the triceps brachii [28,29,30]. Following three PBS/Triton-X100 washes, sections were then incubated for 1 h with a secondary antibody cocktail with specific fluorophores/colors directed to each primary antibody. These included MHCIIA/Alexa Fluor 350 (Millipore #A21120), MHCIIB/Alexa Fluor 488 (Millipore #A21042), and Laminin/Alexa Fluor 555 (Millipore #A21434). Overlapping 10x images were captured with an Olympus IX71 fluorescent microscope of the entire cross-section. Individual images were then equally adjusted for background and color levels (Adobe Photoshop CS5) and stitched into a single panoramic of the entire muscle (Microsoft Image Composite Editor). MHC fiber count, fiber area, and minimum Feret diameter were measured with the semiautomatic muscle analysis using segmentation of histology software (SMASH) with pixel size set to 1.0 μm/pixel, minimum fiber size 200 μm, and maximum fiber size 5000 μm [31]. The minimal Feret diameter of a muscle fiber is defined as the closest possible distance between the two parallel tangents of an object (i.e., muscle fiber); it is a measure of muscle fiber size that may complement cross-sectional area (CSA) measures because it is more impervious to error arising from deviations in the angle of tissue sectioning [32].

### 2.4. Western Blotting

The gastrocnemius from each mouse was pulverized on dry ice using a porcelain mortar and pestle and ~25 mg of pulverized tissue was homogenized on ice in 0.3 mL of homogenization buffer containing 1.0 mM NaCl, 1.0% Triton-X, 0.5% sodium deoxycholate, 10 mM Tris-HCl, pH 7.6 with protease and phosphatase inhibitor cocktails (Sigma, St. Louis, MO, USA). After homogenization, the samples were cleared by centrifugation for 15 min at 5000× *g*. The supernatant was removed, and protein concentration was determined using the BCA protein assay (Pierce #23227; Thermo Fisher Scientific Inc., Rockford, IL, USA). Total protein (30 μg) was prepared in Laemmli buffer (Sigma, St. Louis, MO, USA) and subjected to electrophoretic separation by SDS-PAGE on 7.5% acrylamide gels (Bio-Rad, Hercules, CA, USA) as previously described [33]. Following electrophoretic separation, the proteins were transferred to a polyvinylidene difluoride (PVDF) membrane (Millipore Sigma, St. Louis, MO, USA) and stained with 0.1% Ponceau S in 0.5% acetic acid (Sigma, St. Louis, MO, USA) to ensure equal loading and transfer of proteins. Briefly, non-specific binding was blocked with 1% bovine serum albumin (BSA, Sigma, St. Louis, MO, USA) in TBS-T (Tris-buffered saline, 0.1% Tween 20, Sigma, St. Louis, MO, USA) for 1 h at room temperature. Following blocking, membranes were exposed to the following primary antibodies in 1% BSA/TBS-T overnight at 4 °C: phosphorylated Akt^Ser473^ (Cell Signaling; Danver, MA-9271; 1:500); total Akt (Cell Signaling-9272; 1:1000); phosphorylated p70S6K^Thr389^ (Cell Signaling-9234; 1:500); and total p70S6K (Cell Signaling-2708; 1:1000) phosphorylated 4EBP-1^Thr37/46^ (Cell Signaling-9459; 1:1000); and phosphorylated ribosomal protein S6 (rpS6)^Ser235/236^ (Cell Signaling-2211; 1:1000). After primary antibody incubation, membranes were washed three times and then incubated for 1 h with horseradish peroxidase (HRP)-linked IgG anti-rabbit (Cell Signaling-7074; 1:20,000) secondary antibody. Finally, membranes were washed three times and immediately exposed to SuperSignal™ West Dura ECL chemiluminescent detection HRP reagents (Pierce #35071; Thermo Fisher Scientific Inc., Rockford, IL, USA) at a 1:1 ratio for 5 min. Membranes were covered in plastic wrap and exposed to radiographic film. Films were developed in a dark room using Konica Minolta SRX-101A film processor (Konica Minolta, Ramsey, NJ, USA). The densitometry of specific protein target bands was determined using Image J software (NIH, Bethesda, MD, USA). All values obtained from one blot were normalized to the blot average density. Membranes were stripped with Restore Western blot stripping buffer (#21059; Thermo Fisher Scientific Inc., Rockford, IL, USA) for 10 min, washed with TBS-T and re-probed with additional antibodies, as described above. Complete stripping of antibodies was verified by applying the secondary antibody to the blot and developing as described above.

### 2.5. RNA Isolation and Real-Time PCR Analysis

Total cellular RNA was isolated from ~30 mg of pulverized gastrocnemius muscle using a FastRNA Green Kit (MP Bio, Santa Ana, CA, USA) and RNA-containing homogenates were further purified using PureLink RNA mini columns (Life Technologies, Carlsbad, CA, USA). Quantification of RNA was performed on each sample using spectrophotometry by measuring the absorbance at 260 nm using a Nanodrop 1000 (Thermo Scientific, Rockford, IL, USA). RNA purity was determined by a 260:280 nm ratio of ≥2.0 and the absence of organic contamination such as phenol and carbohydrates was verified by a 230:260 nm ratio of ≥1.8. RNA (1 μg), which was reverse transcribed (#4368814; Life Technologies, Carlsbad, CA, USA) and 25 ng of cDNA per reaction was used for real-time PCR with TaqMan gene expression assays (#4331182; Life Technologies, Carlsbad, CA, USA). Levels of *MAFbx* (Mm00499523_m1), *MuRF-1* (Mm01185221_m1), and *myostatin* (Mm01254559_m1) mRNA were analyzed and normalized to TATA box binding protein endogenous control reference mRNA (*Tbp*; Mm01277042_m1) using a Prism 7300 real-time PCR system (Life Technologies, Carlsbad, CA, USA). Target mRNA levels were calculated using the ∆∆C_t_ method and expressed relative to control samples.

### 2.6. Statistical Analysis

Two-way ANOVAs (treatment group and fiber type) were used to test for differences in fiber CSA, fiber minimum Feret diameter, and fiber type percent. One-way ANOVAs were performed to determine whether differences existed between treatment groups in all other outcome measures. Significance was determined, a priori, at a *p*-value of ≤0.05. Statistical analyses were performed with SPSS version 25 (IBM, Armonk, NY, USA).

## 3. Results

### 3.1. Body, Muscle, and Organ Mass

Body mass was determined for each treatment prior to day 1 (D1) and day 28 (D28) of supplementation. No significant differences (*p* > 0.05) were observed in body mass between treatment groups on D1 and D28 (Table 1). No significant differences (*p* > 0.05) were observed in the muscle tissue wet mass/body mass ratio between treatment groups in the soleus, plantaris, gastrocnemius, tibialis anterior, or extensor digitorum longus muscles (Table 2). In addition, no significant differences (*p* > 0.05) were observed in organ tissue wet mass/body mass between treatment groups for the heart, liver, spleen, kidneys, and testes (Table 3).

### 3.2. Muscle Fiber Cross-Sectional Area and Fiber Type

**Figure 1:**
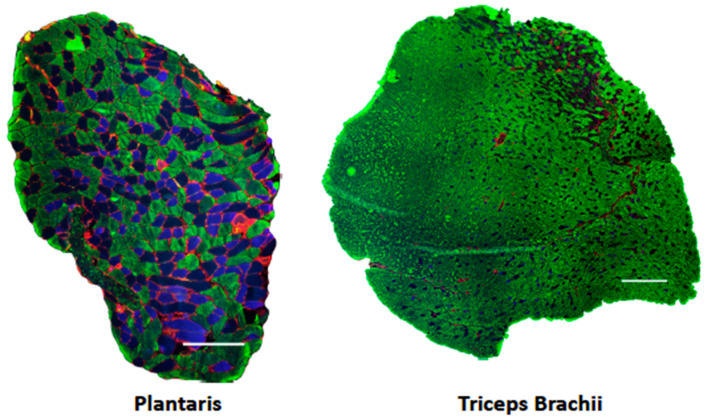
Immunohistochemical analysis of muscle fiber type. Representative images of fiber type and laminin staining on entire plantaris and triceps brachii cross-sections. Blue, type IIa; unstained/black, type IIx; green, type IIb; red, laminin. Scale bar = 200 μm.

Plantaris and triceps brachii muscle cross-sections were visualized using immunohistochemical techniques (*n* = 6–10 samples per group; Figure 1). Results revealed that neither treatment increased fiber diameter, as measured by both CSA and minimum Feret diameter (Table 4). Further, neither treatment affected the proportion of fiber types (Table 4). 

### 3.3. Protein Synthesis Signaling Pathway

**Figure 2:**
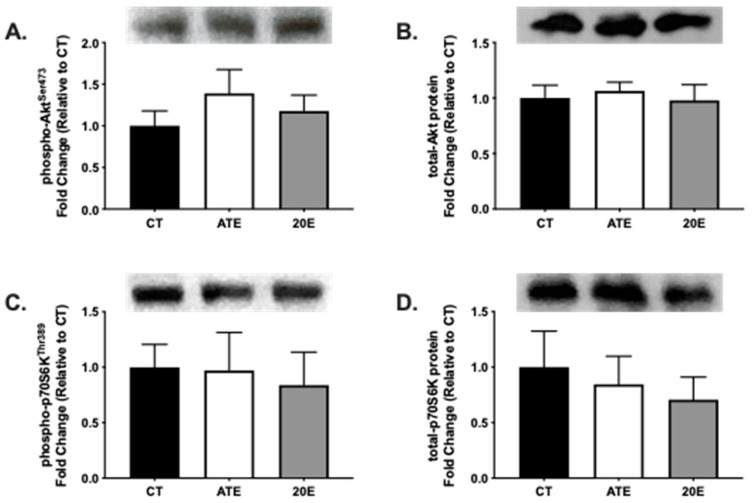
Protein analysis of markers of protein synthesis signaling in aging gastrocnemius muscle via Western blot after the 28-day supplementation period. Quantification of phosphorylated Akt (**A**), total Akt protein (**B**), phosphorylated p70S6K (**C**), and total p70S6K protein (**D**) by treatment, respectively. Representative Western blot protein bands are provided. Data are normalized to CT and presented as mean ± SEM. CT, control; ATE, *A. turkestanica* extract; 20E, 20-hydroxyecdysone. No significant differences were found, *p* > 0.05, all.

Markers for protein synthesis pathway signaling activity and phosphorylation status of Akt and p70S6K, were measured by Western blot analysis in the gastrocnemius muscle (*n* = 6–7 per group) after the 28-day supplementation period. No significant differences were measured in phosphorylation status of Akt^Ser473^ (*p* = 0.485; Figure 2A) or p70S6k^Thr389^ (*p* = 0.914; Figure 2C). In addition, no significant differences were found in total Akt and *p*-70S6K protein expression (Figure 2B,D, respectively) between treatment groups after the 28-day supplementation period (*p* = 0.863 and *p* = 0.194, respectively).

**Figure 3:**
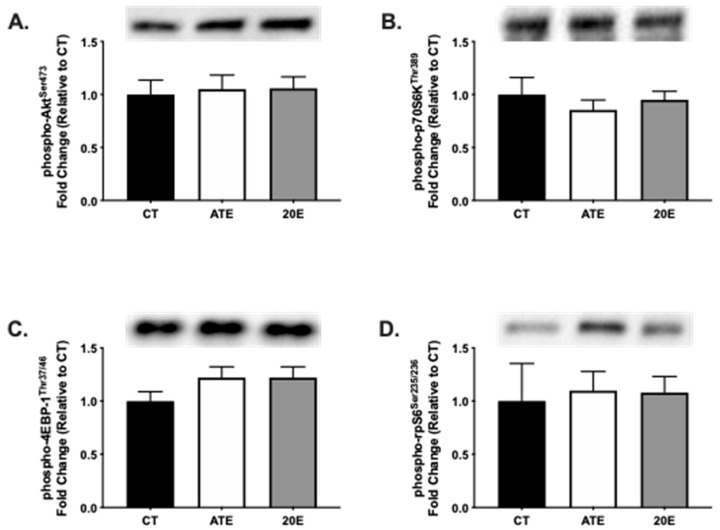
Protein analysis of markers of protein synthesis signaling in aging gastrocnemius muscle via Western blot after an acute dose of supplementation. Quantification of phosphorylated Akt (**A**), phosphorylated p70S6K (**B**), phosphorylated 4EBP-1 (**C**), and phosphorylated rpS6 (**D**) protein by treatment, respectively. Representative Western blot protein bands are provided. Data are normalized to CT and presented as mean ± SEM. CT, control; ATE, *A. turkestanica* extract; 20E, 20-hydroxyecdysone. No significant differences were found, *p* > 0.05, all.

Furthermore, several molecules involved in the activation of the protein synthesis signaling pathway were measured in the gastrocnemius muscle after an acute treatment dose of either ATE, 20E, or vehicle control. There were no significant differences in the phosphorylation status of Akt (*p* = 0.943; Figure 3A), p70S6K (*p* = 0.653; Figure 3B), 4EBP-1 (*p* = 0.234; Figure 3C), or rpS6 (*p* = 0.953; Figure 3D) between treatment groups after an acute treatment dose.

### 3.4. Atrogene and Myostatin mRNA Levels

**Figure 4:**
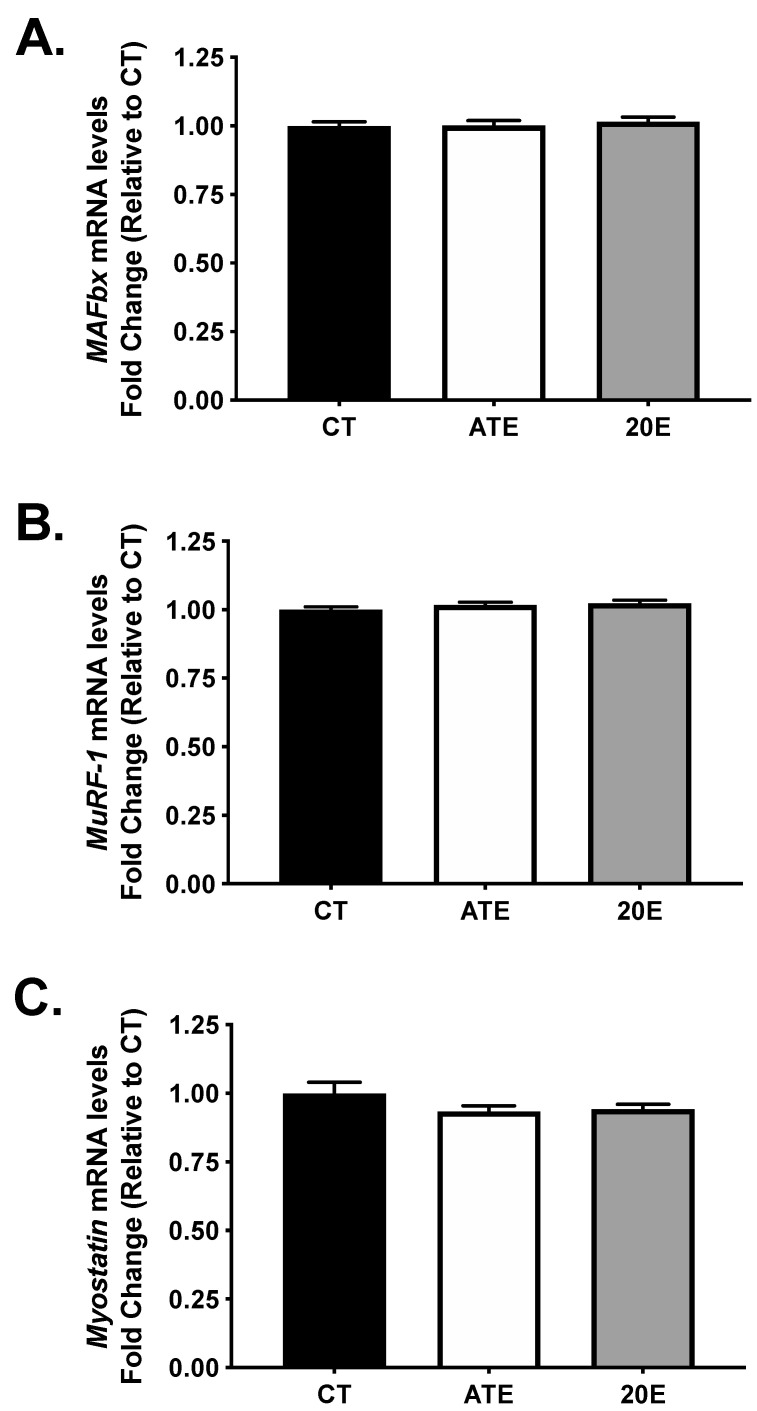
Analysis of mRNA levels for the atrogenes, *MAFBx* and *MuRF-1*, and the negative regulator of muscle mass, *myostatin* in aging gastrocnemius muscle via real-time PCR after the 28-day supplementation period. Quantification of *MAFBx* (**A**), *MuRF-1* (**B**), and *myostatin* (**C**) mRNA levels by treatment, respectively. Data are normalized to CT and presented as mean ± SEM. CT, control; ATE, *A. turkestanica* extract; 20E, 20-hydroxyecdysone. No significant differences were found, *p* > 0.05, all.

Levels of mRNA for the atrogenes, *MAFbx* and *MuRF-1*, as well as the negative regulator of muscle mass, *myostatin*, were measured in the gastrocnemius muscle using real-time PCR (*n* = 11–12 per group) after the 28-day supplementation period. No significant differences were measured in *MAFbx* mRNA (*p* = 0.979; Figure 4A), *MuRF-1* mRNA (*p* = 0.308; Figure 4B), or *myostatin* mRNA (*p* = 0.211; Figure 4C) between treatment groups after the 28-day supplementation period.

## 4. Discussion

This study determined no significant effects of acute or 28-day feeding of ATE and 20E to sedentary aging mice on muscle mass hypertrophy (mass and cross-sectional area), activation of the PI3K–Akt pathway, which is a key control point of protein synthesis, and attenuation of the expression of two E3 ligases regulated by Akt and myostatin.

### 4.1. Phytoecdysteroids in Aging Muscle

The anabolic effects of 20E supplementation have been reported in previous papers [17,18,19,20,22,34,35,36]. Studies do not support a consistent effect of 20E supplementation on gains in muscle and body mass, which may in part be related to differences in 20E dosing regimens (route, dose, duration) and the types of rodents used (muscle condition, and strain/species) [22,36,37]. Positive anabolic effects of phytoecdysteroids have only been reported in young growing (i.e., <3-months of age) and young adult (i.e., +3-months of age) rodents [20,22]. This is the first study to test the effects of acute and chronic phytoecdysteroid supplementation on skeletal muscle taken from sedentary aging mice.

Muscle wet mass, fiber cross-sectional area, and minimum Feret diameter were not altered by either ATE or 20E in plantaris or triceps brachii muscles of sedentary aging mice. Our findings differ from the findings of Tóth et al. [36] and Cheng et al. [22], who reported significant increases in extensor digitorum longus and soleus muscle fiber CSA when young adult rats were administered daily subcutaneous injections of 20E for 8 days and triceps brachii mass after 5 days of continuous infusion, respectively. Additionally, Tóth et al. [36] demonstrated that 20E increased fiber size in a muscle-specific manner depending on the metabolic disposition of the muscle, with type I/IIa and IIx demonstrating the greatest gains in CSA in soleus and extensor digitorum longus muscles, respectively. The route of administration may play a key role in the physiological effects of phytoecdysteroids because subcutaneous injection and continuous infusion of 20E bypasses the typical transformation that occurs in the gastrointestinal tract following oral intake. While phytoecdysteroids are rapidly and extensively metabolized [38,39], the physiological effects of the biotransformed metabolites on skeletal muscle are not completely defined. However, large doses of poststerone, a major metabolite of 20E, have been found to increase muscle fiber CSA after subcutaneous injection near the muscle [40]. Thus, the skeletal muscle results of studies using different routes of administration for phytoecdysteroids cannot be directly compared.

The mechanisms for decreases in muscle fiber size with age are believed to be related to decreases in anabolic/protein synthesis signaling (i.e., PI3K–Akt signaling) and transitions from faster, larger fiber subtypes (i.e., type IIx and IIb) to slower, smaller fiber subtypes (i.e., type IIa). To assess PI3K–Akt signaling with phytoecdysteroid supplementation, upstream (i.e., Akt^Ser473^) and downstream (i.e., p70S6K^Thr389^) markers for this pathway were analyzed in the gastrocnemius. While 20E and ATE have been reported to work through the PI3K–Akt pathway in cultured muscle cells [20,34], we did not observe differences in phosphorylation of either Akt^Ser473^ or p70S6K^Thr389^ between treatments with 28 days of supplementation. The lack of PI3K–Akt signaling activation with chronic treatment may have been due to the oral route of administration or the avoidance of phytoecdysteroid supplementation on the day of sacrifice (i.e., Day 29). Muscle protein synthesis signaling through the PI3K–Akt pathway has been shown to peak within 45–90 min following oral feeding of the amino acid leucine, an activator of the PI3K–Akt pathway, in young animals [41]. Moreover, 20E has been shown to be eliminated rapidly in young adult mice. Dzhukharova et al. [42] reported that a 50 mg/kg caudal vein infusion of [^3^H] 20E was ~90% eliminated within 30 min of treatment. The authors also reported that 20E has a half-life of ~8 min. Thus, we proceeded with an acute treatment experiment in which aging mice were provided an acute oral dose of 50 mg/kg ATE or 20E to assess acute PI3K–Akt signaling activation. We were still unable to observe activation of the PI3K–Akt signaling pathway at 90 min post-acute treatment with ATE or 20E. Our finding is supported by Anthony et al. [43], who reported that acute intake of 20E did not stimulate the PI3k–Akt pathway in young growing rats.

Akt phosphorylates forkhead box-O protein (FOXO). When phosphorylated, FOXO cannot translocate to the nucleus and increase the expression of key elements of the ubiquitin–proteasome pathway that include MAFbx and MuRF-1 [8]. Myostatin is a member of the transforming growth factor β (TGFβ) superfamily, and when overexpressed, protein synthesis is inhibited by downregulating the activity of the PI3K–Akt pathway [5,44]. Blocking myostatin in aging mice increases muscle mass and improves muscle function [15]. Treating C2C12 myotubes with ATE has been reported to significantly decrease *myostatin* mRNA levels [21]. In this study, chronic oral intake of ATE or 20E did not alter *MAFbx*, *MuRF-1*, or *myostatin* mRNA levels in the gastrocnemius.

### 4.2. Method for Dietary Phytoecdysteroid Supplementation

To minimize distress and potential injury to the animals, the mice were allowed to voluntarily consume ATE, 20E, or CT in a 190 mg food pellet. This method was well accepted by the mice and could be considered in future chronic feeding studies to minimize stress and injury in aged rodents. 

### 4.3. Study Limitations

The dose of 50 mg/kg was selected based on the study published by Gorelick-Feldman et al. [20] supporting an increase in grip strength in young growing rats after 28 days of treatment. Our acute treatment protocol and data are consistent with those of Anthony et al. [43], who measured PI3K–Akt signaling within 30 min of treatment. Future studies could use a shorter time interval of 10–20 min. Another limitation of the current study is that the mice were sedentary. The anabolic effect of phytoecdysteroids may be realized when the skeletal muscle is placed under a stress, such as the skeletal muscle regeneration model reported by Tóth et al. [36].

## 5. Conclusions

This is the first study to examine the effects of acute and chronic oral intake of phytoecdysteroids on skeletal muscle outcomes in sedentary aging male mice. Intake of the phytoecdysteroid mixture (*A. turkestanica* extract) or the isolated phytoecdysteroid (20-hydroxyecdysone) did not alter muscle fiber cross sectional area, anabolic signaling, or inhibitors of skeletal muscle mass. These null results could be due to several factors including the oral route of phytoecdysteroid administration and subsequent metabolic transformation, the advanced age of the male mice, and the research design model that did not incorporate concurrent stress on the muscle.

## Figures and Tables

**Table 1:** Animal body mass by group pre- and post-28-day supplementation period.

Treatment	Body Mass D1 (g)	Body Mass D28 (g)	*p*-Value
CT	32.7 ± 1.0	32.4 ± 1.1	0.44
ATE	33.0 ± 0.9	32.8 ± 0.7	0.40
20E	33.4 ± 0.4	32.5 ± 0.6	0.12

Data are presented as mean ± SEM; D1, day 1; D28, day 28; CT, control; ATE, *Ajuga turkestanica* extract; 20E, 20-hydroxyecdysone. No significant differences were found, *p* > 0.05, all.

**Table 2:** Hindlimb muscle wet weight.

Muscle (mg/g BM)	CT	ATE	20E	*p*-Value
Soleus	0.27 ± 0.00	0.27 ± 0.01	0.28 ± 0.00	0.36
Plantaris	0.62 ± 0.01	0.60 ± 0.01	0.65 ± 0.02	0.27
Gastrocnemius	4.13 ± 0.13	3.96 ± 0.11	4.07 ± 0.11	0.60
Tibialis Anterior	1.74 ± 0.08	1.69 ± 0.03	1.74 ± 0.03	0.72
Extensor Digitorum Longus	0.39 ± 0.02	0.36 ± 0.01	0.36 ± 0.10	0.50

Data are presented as mean ± SEM; mg/g BM, mg tissue/g body mass; CT, control; ATE, *A. turkestanica* extract; 20E, 20-hydroxyecdysone. No significant differences were found, *p* > 0.05, all.

**Table 3:** Organ wet weight.

Organ (mg/g BM)	CT	ATE	20E	*p*-Value
Heart	5.0 ± 0.2	4.8 ± 0.2	4.9 ± 0.1	0.66
Liver	46.4 ± 3.6	40.4 ± 0.9	41.6 ± 1.2	0.16
Spleen	3.8 ± 1.4	2.4 ± 0.2	2.6 ± 0.1	0.47
Kidneys	13.6 ± 0.4	14.1 ± 0.5	14.5 ± 0.5	0.44
Testes	5.7 ± 0.2	5.5 ± 0.1	5.4 ± 0.1	0.57

Data are presented as mean ± SEM; mg/g BM, mg tissue/g body mass; CT, control; ATE, *A. turkestanica* extract; 20E, 20-hydroxyecdysone. No significant differences were found, *p* > 0.05, all.

**Table 4:** Plantaris and triceps brachii morphology.

		Plantaris				Triceps			
		CT	ATE	20E	*p*-Value	CT	ATE	20E	*p*-Value
Fiber CSA (μm)	IIa	1000 ± 77	928 ± 56	1163 ± 149	0.720	926 ± 62	832 ± 46	837 ± 31	0.711
	IIx	1840 ± 126	1875 ± 88	1751 ± 137		1416 ± 76	1305 ± 74	1387 ± 68	
	IIb	2407 ± 149	2482 ± 29	2365 ± 208		2506 ± 84	2554 ± 88	2572 ± 77	
Min. Feret Dia.	IIa	30.2 ± 1.17	29.4 ± 0.96	32.1 ± 2.07	0.656	28.9 ± 0.94	27.7 ± 0.74	28.1 ± 0.52	0.848
	IIx	41.1 ± 1.50	42.2 ± 1.02	40.1 ± 1.71		36.2 ± 0.94	35.1 ± 1.02	36.2 ± 0.81	
	IIb	48.0 ± 1.56	48.9 ± 0.30	46.8 ± 2.42		49.0 ± 0.96	49.4 ± 0.87	49.5 ± 0.68	
Fiber Type (%)	IIa	32.2 ± 1.99	32.3 ± 2.87	27.9 ± 3.88	0.507	8.66 ± 1.29	10.67 ± 4.21	7.84 ± 1.1	0.787
	IIx	20.7 ± 1.68	18.7 ± 2.55	23.9 ± 1.93		14.2 ± 2.10	17.7 ± 5.48	15.5 ± 2.97	
	IIb	47.2 ± 1.51	49.0 ± 1.76	48.1 ± 1.51		77.1 ± 2.22	71.6 ± 9.57	76.7 ± 3.70	

Data are presented as mean ± SEM. CT, control; ATE, *A. turkestanica* extract; 20E, 20-hydroxyecdysone; CSA, cross-sectional area; Minimum Feret Diameter (Min. Feret Diameter); IIa, myosin heavy chain IIa; IIx, myosin heavy chain IIx; IIb, myosin heavy chain IIb. The *p*-value represents the treatment group × fiber type interaction effect. No significant differences were found, *p* > 0.05, all.

## Supplementary Materials

Supplementary materials are available online at https://www.mdpi.com/1660-4601/18/2/370/s1, Figure S1: The first extraction of the 6% “crude” *A. turkestanica* extract was suspended in water and partitioned with ethyl acetate (EthAc). The ethyl acetate extract (6.27 g) was dried and set aside. The remaining aqueous layer (AQ) was then extracted with n-butanol (BuOH). The BuOH extract (35.13 g) was split and half of the extract (17.57 g) was extracted with EthAc in 0.5% methanol (MeOH). The 0.5% MeOH in EthAc extract (2.47 g) was then combined with 1.64 g of the remaining half of BuOH extract at a 2:3 EthAc extract to BuOH extract ratio (i.e., 2:3 mixture) to obtain a 1:4 20E:turkesterone ratio. The 2:3 mixture was then extracted first with EthAc and then BuOH. The EthAc extract (1.65 g) was dried and combined with the dried BuOH extract (0.99 g) to make a 1:4 20E to turkesterone ratio. TLC analysis of the 1:4 *A. turkestanica* extract (ATE; 1.31 g) revealed that the major phytoecdysteroid compounds were present in the extract. However, the amount of ATE was not sufficient for 28 days of supplementation, and a second extraction of 100 g was required. Figure S2: The second extraction of 6% “crude” *A. turkestanica* extract was solubilized in and extracted with ethyl acetate (EthAc). The EthAc extract (6.79 g) was dried and set aside. The remaining aqueous layer was then partitioned with butanol (BuOH). The dried BuOH extract was then extracted with 0.2% methanol (MeOH) in EthAc. The 0.2% MeOH in EthAc extract (1.02 g) was then combined with the EthAc extract (1.56 g) and the 1st extraction 1:4 mixture (1.31 g) to create a final 1:4 ATE (3.89 g). TLC analysis of the final 1:4 ATE revealed that the major phytoecdysteroid compounds were present in the extract. Figure S3: HPLC chromatogram of the final 1:4 *A. turkestanica* extract (ATE). Peak identities were recorded at a wavelength of 247 nm for the identified phytoecdysteroids: turkesterone (*R_t_* 22.7 min), 20E (*R_t_* 29.6 min), cyasterone (*R_t_* 36.7 min), ajugasterone (*R_t_* 40.3 min), ajugalactone (*R_t_* 50.0 min), and cyasterone 22-acetate (*R_t_* 51.9 min). Figure S4: ATE and 20E phytoecdysteroid content. The commercial 20E (i.e., 80.11% total phytoecdysteroid) utilized for the supplementation protocol was different than the purified commercial 20E (i.e., 100% total phytoecdysteroid) utilized for the phytoecdysteroid quantification calibration curve. The identified phytoecdysteroids contained within the 1:4 *A. turkestanica* extract (ATE) dry weight include turkesterone (5.85%), 20E (23.61%), cyasterone (3.07%), ajugasterone (2.02%), ajugalactone (3.98%), and cyasterone 22-acetate (2.23%) for a total of 40.77% total phytoecdysteroid. The commercial 20E used for supplementation contained 80.11% total phytoecdysteroid based on dry weight, determined by HPLC.
