# Supplementary material for: Phytoecdysteroids Do Not Have Anabolic Effects in Skeletal Muscle in Sedentary Aging Mice
Source: Int J Environ Res Public Health. 2021 Jan 6;18(2):370. doi: 10.3390/ijerph18020370 (PMC7825148; doi:10.3390/ijerph18020370)
Supplement: Supplementary file 1 [file ijerph-18-00370-s001.pdf]

Article

Phytoecdysteroids Do Not Have Anabolic Effects in Skeletal Muscle in Sedentary Aging Mice

Marcus M. Lawrence [1,2,3,4,5], Kevin A. Zwetsloot [1,3,6], Susan T. Arthur [5], Chase A. Sherman [1,3], Joshua R. Huot [5], Vladimir Badmaev [7], Mary Grace [8], Mary Ann Lila [8], David C. Nieman [2,6] and R. Andrew Shanely [1,2,3,*]

[1] Department of Health and Exercise Science, Appalachian State University, Boone, NC 28608, USA; marcuslawrence@suu.edu (M.M.L.); zwetslootka@appstate.edu (K.A.Z.); casherman0412@email.campbell.edu (C.A.S.)
[2] Human Performance Lab, North Carolina Research Campus, Kannapolis, NC 28081, USA; niemandc@appstate.edu
[3] Integrated Muscle Physiology Laboratory, Boone, NC 28607, USA
[4] Department of Kinesiology and Outdoor Recreation, Southern Utah University, Cedar City, UT 84720, USA
[5] Laboratory of Systems Physiology, Department of Kinesiology, University of North Carolina Charlotte, Charlotte, NC 28223, USA; sarthur8@uncc.edu (S.T.A.); jrhuot@iu.edu (J.R.H.)
[6] Department of Biology, Appalachian State University, Boone, NC 20608, USA
[7] American Medical Holdings Inc., Staten Island, NY 10314, USA; vebadmaev@attglobal.net
[8] Plants for Human Health Institute, North Carolina Research Campus, North Carolina State University, Kannapolis, NC 28081, USA; mhgrace@ncsu.edu (M.G.); maryann_lila@ncsu.edu (M.A.L.)
* Correspondence: shanelyra@appstate.edu; Tel.: +1-828-262-6319

Plant Materials and Crude Extraction

Whole *Ajuga turkestanica* plant material was harvested in Las Palmas, Spain (PoliNat; Las Palmas, Spain). The plant material was air dried and milled to 1-2 mm particle size. A crude extraction consisting of a 30:70 water-ethanol solvent solution at 60°C to extract phytoecdysteroids and sterilize the plant material was performed by P.L. Thomas Inc. (Morristown, NJ); P.L. Thomas Inc. kindly donated this initial extract. HPLC analysis of the sterilized and extracted *A. turkestanica* plant material, normalized to a 20E standard, revealed a 6% mg/mL total phytoecdysteroid content in the initial crude extract. The initial extract was air dried and sieved through a 60 mm mesh screen and stored at -20°C until further extraction and analysis.

Phytoecdysteroid Enrichment and Standardization

The extract enriched in phytoecdysteroids from the initial *A. turkestanica* extract (ATE) was performed using established methods (Cheng, 2008), with modifications. Briefly, (1st extraction) 100 grams of the initial crude extract was suspended in 500 mL of sterile ddH$_2$O and sonicated for 10 minutes. The suspension was defatted by partition (liquid-liquid extraction) with n-hexane (500 mL x 4). The defatted aqueous layer was then partitioned with ethyl acetate (500 mL x 8). The combined ethyl acetate extract was dried over anhydrous sod-sulfate and the solvent was removed via rotary evaporation to produce the ethyl acetate extract. The dried ethyl acetate extract was analyzed by TLC silica gel 60 F$_{254}$ 250 μm pre-coated plates (EMD Chemicals Inc., Gibbstown, NJ) with a solvent system of ethyl acetate-methanol-water at 77:13:19 ratio. The TLC plates were monitored by short wave UV lamps (254 nm) and visualized with vanillin reagent (4 g vanillin, 100 mL methanol and 2.5 mL H_2SO_4) (Stahl, 1969). Following identification of phytoecdysteroids by TLC visualization, HPLC analysis was performed for quantification of phytoecdysteroids. A commercial HPLC grade standard of 20E (Sigma, St. Louis, MO) at concentrations of 250, 500 and 1000 μg/ml with 10 μL injection volumes was used to determine the concentrations of phytoecdysteroids in the extract. Analysis was performed using an Agilant 1200 HPLC system (Agilent Technologies, Inc., Wilmington, DE) with autosampler, DAD (247 nm) and Synergi 4 um Hydro-RP 80A reversed phase column (250 mM x 4.6 mm x 5 um, Phenomenex, Torrance, CA). The mobile phase solvents consisted

of 2% acetic acid in H_2O (solvent A) and 0.5% acetic acid in 50% aqueous acetonitrile (solvent B). A gradient of 10%, 15%, 25%, 35%, 55%, 100%, and 10% solvent B was used at 0, 10, 13, 20, 50, 54 and 60 min, respectively, with 1.0 mL/min flow rate. Samples were filtered through 0.2 mm PTFE filters before injecting 10 µl on the HPLC column (25°C). Identification of phytoecdysteroids was performed in reference to compounds isolated previously from *A. turkestanica* (Cheng, 2008). Quantification of compounds was performed from the peak areas recorded at 247 nm for phytoecdysteroids in reference to the calibration curve obtained with the purified commercial 20E standard (i.e., 100% total phytoecdysteroids). Concentrations were calculated by taking the average of two HPLC runs.

The aqueous layer remaining after the ethyl acetate partition was then partitioned with butanol (750 mL x 3). Butanol was removed via rotary evaporation and the residue obtained was freeze-dried to produce the butanol extract. Following identification of phytoecdysteroids by TLC visualization, HPLC analysis was performed for quantification of phytoecdysteroids. The aqueous layer after butanol extraction was discarded. The dried butanol extract was pulverized with a porcelain mortar and pestle, heated in 60°C water bath and sonicated for 10 minutes to help dissolution, and then vacuum filtered through #4 filter paper (Whatman International Ltd., England). One-half of the dried butanol extract was then extracted with 0.5% methanol in ethyl acetate (100 mL x 4). The solvent was removed via rotary evaporation and the combined ethyl acetate extracts was dehydrated over anhydrous sod-sulfate and lyophilized. Based on HPLC results, the 0.5% methanol in ethyl acetate dried extract was then combined with the remaining one-half dried butanol extract in a 2:3 ratio. The 2:3 mixture was first extracted with methanol (100 mL x 4) and then butanol (100 mL x 4). The solvents from the 2:3 extract were rotatory evaporated and lyophilized. From the concentrations determined by HPLC, the dried methanol 2:3 mixture extract and butanol 2:3 mixture extract were then combined to create a 1:4 ratio of 20E to turkesterone; the final *A. turkestanica* extract (ATE). The 1:4 ATE was analyzed by TLC as described above and then analyzed for phytoecdysteroid content by HPLC. Two rounds of extraction were necessary to produce a sufficient amount of ATE for this study (Figure 1).

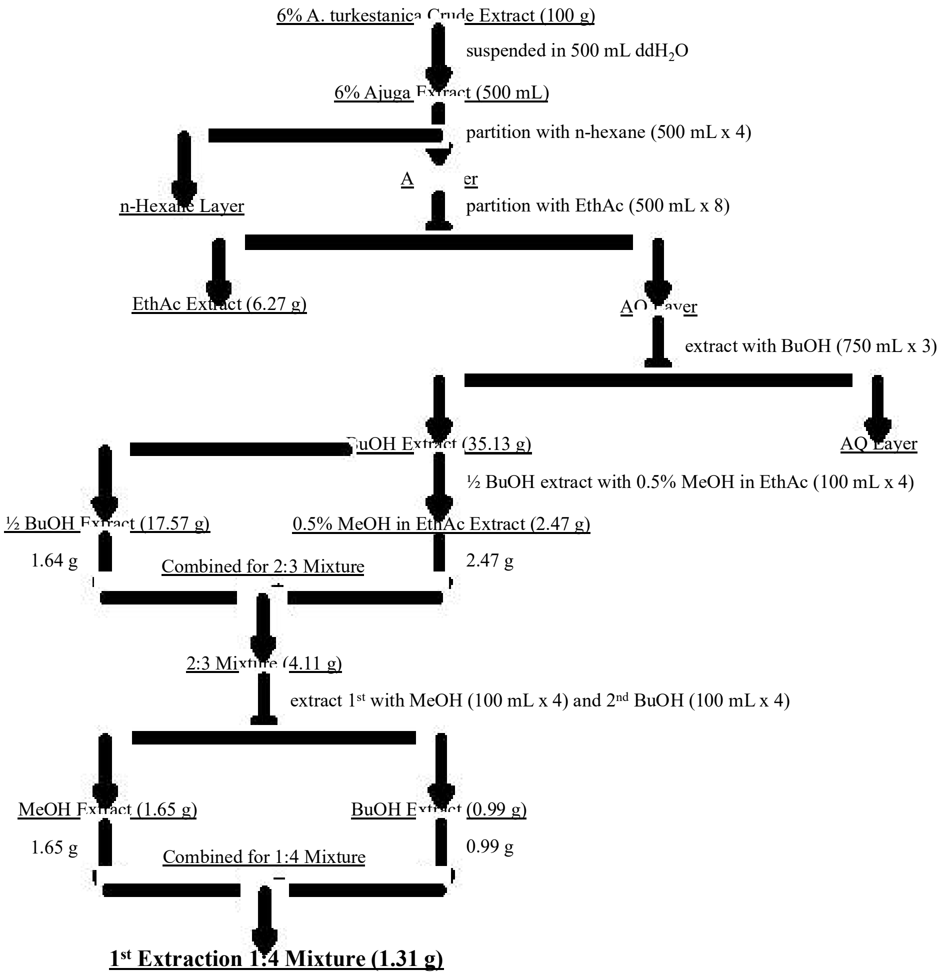

Figure 1. The first extraction of the 6% "crude" *A. turkestanica* extract was suspended in water and partitioned with ethyl acetate (EthAc). The ethyl acetate extract (6.27 g) was dried and set aside. The remaining aqueous layer (AQ) was then extracted with n-butanol (BuOH). The BuOH extract (35.13 g) was split and ½ of the extract (17.57 g) was extracted with EthAc in 0.5% methanol (MeOH). The 0.5% MeOH in EthAc extract (2.47 g) was then combined with 1.64 g of the remaining ½ BuOH extract at 2:3 EthAc extract to BuOH extract ratio (i.e. 2:3 mixture) to obtain a 1:4 20E:turkesterone ratio. The 2:3 mixture was then extracted first with EthAc and then BuOH. The EthAc extract (1.65 g) was dried and combined with the dried BuOH extract (0.99 g) to make a 1:4 20E to turkesterone ratio. TLC analysis of the 1:4 *A. turkestanica* extract (ATE; 1.31 g) revealed that the major phytoecdysteroid compounds were present in the extract. However, the amount of ATE was not sufficient for 28 days of supplementation and a second extraction of 100 g was required.

The 2nd extraction was simplified based on the results obtained from the 1st batch extraction. Briefly, 100 g of the initial *A. turkestanica* extract was suspended in 500 mL of sterile double distilled water (ddH2O) and sonicated for 10 minutes. The suspension was then partitioned with ethyl acetate (500 mL x 10). The ethyl acetate was dried over anhydrous sod-sulfate and the solvent was removed via rotary evaporation and the residue was lyophilized. The remaining aqueous layer was then extracted with butanol (500 mL x 5). The butanol was removed via rotary evaporation and remaining residue was lyophilized. The dried butanol extract was pulverized with a porcelain mortar and pestle, heated in 60°C water bath and sonicated for 10 minutes to help dissolution and then vacuum filtered through #4 filter paper (Whatman International Ltd., England). The dried butanol extract was then extracted with 0.2% methanol in ethyl acetate (100 mL x 4). The 0.2% methanol in ethyl acetate extract was dried over anhydrous sod-sulfate and the solvents were removed via rotary evaporation and the residue was lyophilized. From the concentrations determined by HPLC, the dried 0.2% methanol in ethyl acetate was then combined with the dried ethyl acetate and the 1:4 ATE from extraction 1 to create the final 1:4

ATE that was used for the supplementation protocol (Figure 2). The final 1:4 ATE was analyzed by TLC as described above and then analyzed for phytoecdysteroid content by HPLC.

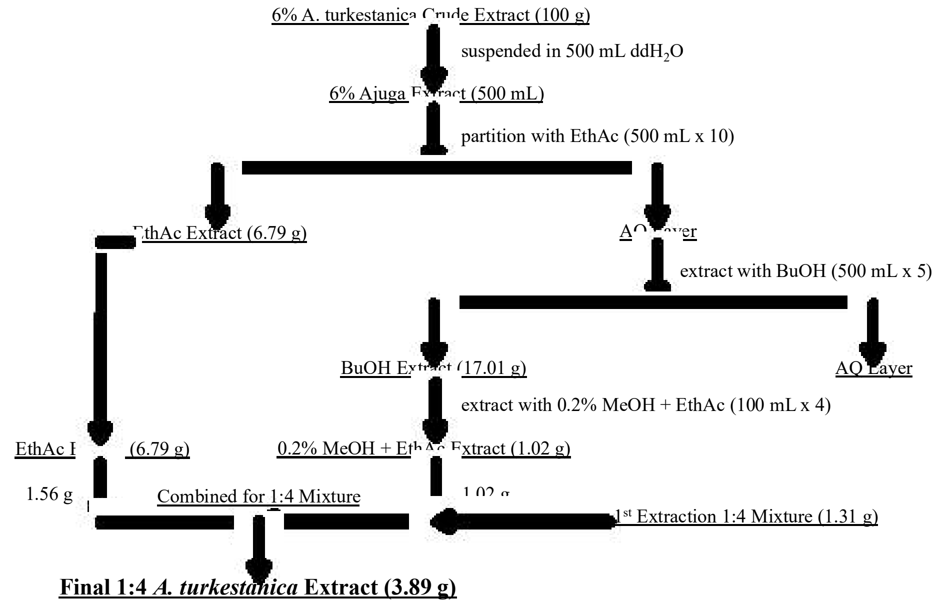

Figure 2. The second extraction of 6% "crude" *A. turkestanica* extract was solubilized in and extracted with ethyl acetate (EthAc). The EthAc extract (6.79 g) was dried and set aside. The remaining aqueous layer was then partitioned with butanol (BuOH). The dried BuOH extract was then extracted with 0.2% methanol (MeOH) in EthAc. The 0.2% MeOH in EthAc extract (1.02 g) was then combined with the EthAc extract (1.56 g) and the 1st extraction 1:4 mixture (1.31 g) to create a final 1:4 ATE (3.89 g). TLC analysis of the final 1:4 ATE revealed that the major phytoecdysteroid compounds were present in the extract.

Isolation of phytoecdysteroids from *A. turkestanica*

The 200g (100 g x 2) ethyl acetate, butanol and methanolic extraction of initial dried *A. turkestanica* plant material yielded a 3.89 g enriched phytoecdysteroid extract. TLC analysis of the final 1:4 ATE revealed that the major phytoecdysteroid compounds were present in the extract. Peak identities were recorded at a wavelength of 247nm for the identified phytoecdysteroids: turkesterone (R_t 22.7 min), 20E (R_t 29.6 min), cyasterone (R_t 36.7 min), ajugasterone (R_t 40.3 min), ajugalactone (R_t 50.0 min) and cyasterone 22-acetate (R_t 51.9 min), determined by HPLC (Figure 3).

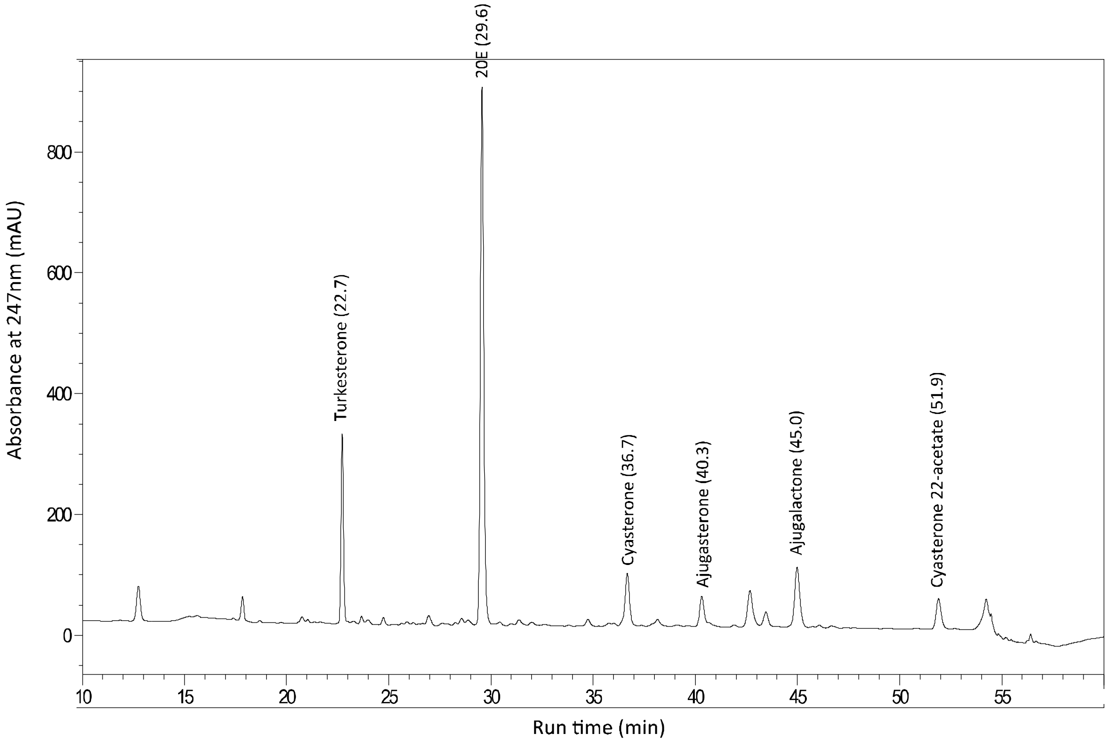

Figure 3. HPLC chromatogram of the final 1:4 *A. turkestanica* extract (ATE). Peak identities were recorded at a wavelength of 247nm for the identified phytoecdysteroids: turkesterone (R_t 22.7 min), 20E (R_t 29.6 min), cyasterone (R_t 36.7 min), ajugasterone (R_t 40.3 min), ajugalactone (R_t 50.0 min) and cyasterone 22-acetate (R_t 51.9 min).

These results are consistent with the commercial standards. The identified phytoecdysteroids contained within the 1:4 ATE dry weight include: turkesterone (5.85%, 0.29 mg/mL), 20E (23.61%, 1.18 mg/mL), cyasterone (3.07%, 0.15 mg/mL), ajugasterone (2.02%, 0.10 mg/mL), ajugalactone (3.98%, 0.20 mg/mL) and cyasterone 22-acetate (2.23%, 0.11 mg/mL) (Figure 4). Peak identity of the commercial 20E used for supplementation occurred at R_t 29.4 min and contained 80.11% total phytoecdysteroid and 0.4 mg/mL dry weight, determined by HPLC (Figure 4).

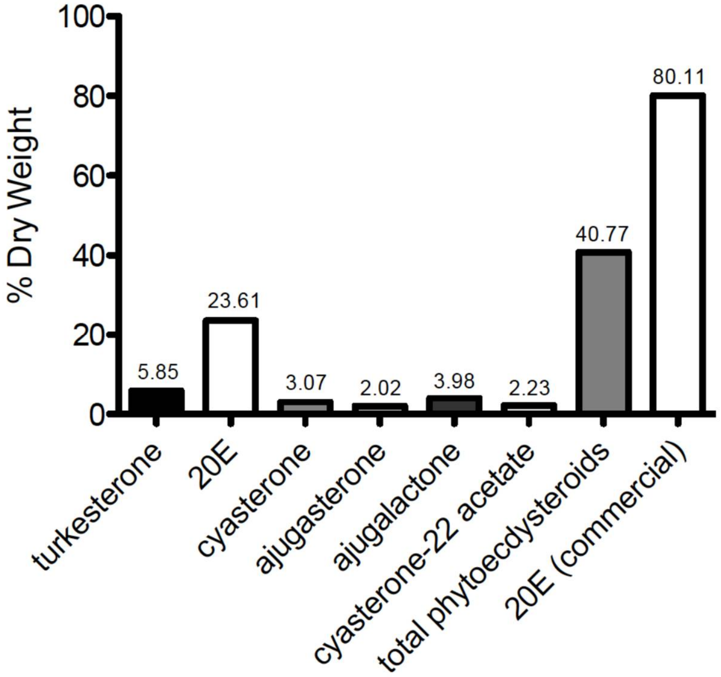

Figure 4. ATE and 20E Phytoecdysteroid Content. The commercial 20E (i.e. 80.11% total phytoecdysteroid) utilized for the supplementation protocol was different than the purified commercial 20E (i.e. 100% total phytoecdysteroid) utilized for the phytoecdysteroid quantification calibration curve. The identified phytoecdysteroids contained within the 1:4 *A. turkestanica* extract (ATE) dry weight include: turkesterone (5.85%), 20E (23.61%), cyasterone (3.07%), ajugasterone (2.02%), ajugalactone (3.98%) and cyasterone 22-acetate (2.23%) for a total of 40.77% total phytoecdysteroid. The commercial 20E used for supplementation contained 80.11% total phytoecdysteroid based on dry weight, determined by HPLC.

References

Cheng, D., Yousef GG, Grace MH, Rogers RB, Gorelick-Feldman J, Raskin I, Lila MA, 2008. In vitro production of metabolism-enhancing phytoecdysteroids from Ajuga turkestanica. Plant Cell Tiss Organ Cult 93, 73-83.

Stahl, E., 1969. [Thin layer chromatography for characterization of pharmacopeia drugs. 5. Chamomile flowers flores Chamomillae]. Arzneimittelforschung 19, 1892-1895.
